# The Combined Application of Intra-Articular Platelet-Rich Plasma Injections and Photobiomodulation Improves Clinical Outcomes in Dogs with Osteoarthritis—Results of a Long-Term, Double-Blinded, Crossover Study

**DOI:** 10.3390/vetsci12111025

**Published:** 2025-10-23

**Authors:** J. C. Alves, Ana Santos, L. Miguel Carreira

**Affiliations:** 1 Divisão de Medicina Veterinária, Guarda Nacional Republicana (GNR), Rua Presidente Arriaga, 9, 1200-771 Lisbon, Portugal; 2 Faculty of Veterinary Medicine, Lusófona University, 1749-024 Lisbon, Portugal; 3 Centro de Ciência Animal e Veterinária, Lusófona University, 1749-024 Lisbon, Portugal; 4 I-MVET, Faculty of Veterinary Medicine, Lusófona University, Lisbon University Centre, 1749-024 Lisbon, Portugal; 5 MED—Mediterranean Institute for Agriculture, Environment and Development, Instituto de Investigação e Formação Avançada, Universidade de Évora, Pólo da Mitra, Ap. 94, 7006-554 Évora, Portugal; 6 Faculty of Veterinary Medicine, University of Lisbon (FMV/ULisboa), 1300-477 Lisbon, Portugal; 7 Interdisciplinary Centre for Research in Animal Health (CIISA), University of Lisbon (FMV/ULisboa), 1300-477 Lisbon, Portugal; 8 Anjos of Assis Veterinary Medicine Centre (CMVAA), 2830-077 Barreiro, Portugal

**Keywords:** dog, osteoarthritis, chronic pain, orthopedics, platelet-rich plasma, regenerative therapy, photobiomodulation

## Abstract

**Simple Summary:**

Osteoarthritis is a very common joint disease in dogs, and clinicians usually favor a multimodal approach for the management of the disease. Recent published information highlighted the fact that the disease is present from a very young age, raising the need for long-term management and concerns around possible medication related side effects. Also, for those reasons, there has been a growing interest around platelet-rich plasma and photobiomodulation, alongside an increasing body of evidence supporting their use. Although there are studies reporting the effect of these treatments individually, there is still a lack of information on their combined use. We aimed to evaluate the effect of the intra-articular administration of platelet-rich plasm, photobiomodulation, or their combined use in dogs with hip osteoarthritis, in a crossover study with a long follow-up period. Our results show that the combination of the two treatments lead to greater, longer-lasting, clinically significant improvements.

**Abstract:**

Thirty dogs were equally assigned to a platelet-rich plasma group (PRPG), a photobiomodulation group (PBMTG), or a combined therapies group (PRP + PBMTG). Response to treatment was evaluated with weight-bearing distribution and different owner-reported outcome measures. Evaluations were conducted at 0, +8, +15, +30, +60, +90, +120, +150, and +180 days after the initial treatment. After the first 180 days, a crossover was performed, and a second 180-day follow-up was conducted. A second cross-over was performed, with a final 180-day follow-up. Nineteen males and eleven females were included, with a mean age of 9.4 ± 2.7 years and a body weight of 26.6 ± 3.8 kg. Six hips were classified as mild, eighteen as moderate, and six as severe. All treatments were able to produce clinically significant improvements in different evaluation modalities, with varied degrees of magnitude and duration. At the last follow-up, the combination of PRP and PBMT had a greater effect, showing a significant difference compared to the isolated treatments, with a moderate to large effect size. Kaplan–Meier estimators showed that PRP + PBMTG had more extended periods with better results. PRP and PBMT could improve objective outcomes and client-reported outcome measures in dogs with OA. Their combined use leads to greater, longer-lasting, clinically significant improvements.

## 1. Introduction

Osteoarthritis (OA) is the most common orthopedic diagnosis in dogs [1], and the disease significantly impacts the patient’s overall quality of life, as it produces pain and affects joint function and mobility [2,3,4]. These factors—particularly chronic pain and reduced mobility—are associated with an increased risk of euthanasia in affected dogs [5,6]. Traditionally, OA was considered a condition of aging, with the average age of diagnosis ranging from 6 to 8 years [1]. However, in a prospective survey of dogs aged 8 months to 4 years, 39.8% exhibited radiographic signs of OA, and 23.6% showed clinical symptoms [7]. As reflected in the broad number of therapeutic approaches described, disease management is challenging [8,9], with a multimodal approach being preferred by clinicians [10]. With the awareness that more animals show clinical signs at a very young age, the risk for adverse events increases, associated with the long-term administration of medication [11,12]. This concern is true even for medications presented as first-line treatment for OA [13,14], either with NSAIDs [15,16,17] or with more recent classes of medications [18].

For those reasons, approaches based on autologous platelet therapies have gained interest, as platelets are a part of the body’s natural response to injury. When administered intra-articularly, platelet-rich plasma (PRP) can contribute to tissue regeneration by promoting cartilage synthesis or inhibiting its breakdown, reducing apoptosis and inflammation, enhancing extracellular matrix production, and influencing angiogenesis [19,20,21,22,23,24]. There are conflicting reports available on the in vivo effects of platelet products. A possible reason for these findings is the insufficient reporting of experimental details or basic attributes of the formulations delivered, including platelet and leukocyte concentrations (including white blood cell differential), which critically influence the outcome [25]. In reports where the reporting guidelines are respected, a single intra-articular (IA) PRP injection produced clinical improvement lasting 12 weeks or more, with some cases showing no progression in radiographic signs [25,26,27]. Multiple PRP injections have also been described, with improvements in range of motion, pain, lameness, and gait kinetics [28,29]. These benefits, as measured by validated client-reported outcome tools, have been reported to persist for an average of 120 to 150 days [24,30].

Photobiomodulation therapy (PBMT) has garnered increasing interest due to the therapeutic potential of red and near-infrared light in promoting tissue healing, providing analgesia, and reducing inflammation [30]. Similarly to PRP, the variability in reported effects of PBMT in the literature is likely attributable to differences in treatment parameters [31]. In veterinary medicine, PBMT has been used to manage a range of conditions in dogs, including osteoarthritis (OA), gingivostomatitis, wound healing, and even gastrointestinal disorders such as diarrhea [31,32,33,34,35]. The beneficial effects of PBMT in managing osteoarthritis are thought to result from the interaction of delivered photons with cellular components. This interaction leads to the dissociation of inhibitory nitric oxide, enhanced electron transport, and increased ATP production. Additionally, PBMT upregulates the expression of genes involved in protein synthesis—particularly those encoding anti-apoptotic proteins and antioxidant enzymes—thereby promoting cell proliferation and anti-inflammatory signaling [32].

In a preliminary study to determine adequate treatment parameters and frequency, we evaluated the isolated and combined use of IA PRP and PBMT in dogs with bilateral hip OA [24]. PRP and PBMT produced clinically significant improvements compared to the assessment on treatment day, but their combined use lead to greater, longer-lasting, clinically significant improvements [24]. This long-term, double-blinded, crossover study aimed to evaluate the combined treatment with intra-articular PRP and PBMT in dogs with bilateral hip OA. We hypothesized that combining the two treatments would be more effective in alleviating OA-related clinical signs than their use as a single modality.

## 2. Materials and Methods

The study protocol was approved by the ethical review committee of the University of Évora (Órgão Responsável pelo Bem-Estar dos Animais da Universidade de Évora, approval nº GD/16901/2022). All methods were carried out in accordance with relevant guidelines and regulations, adhering to the ARRIVE guidelines. Informed consent and permission were obtained in writing from the Institution responsible for the animals (Guarda Nacional Republicana, Portuguese Gendarmerie).

The sample size of the study was determined before the onset of the study by conducting a statistical power analysis (type-1 error, 0.05; type-2 error, 0.8) to determine the minimal number of dogs necessary to perform statistical comparisons between study groups. Thirty dogs diagnosed with bilateral hip OA were enrolled in the study. All animals presented a consistent clinical history, with handlers reporting specific signs such as difficulty rising, jumping, and maintaining obedience positions. On physical examination, pain during joint mobilization, stiffness, and reduced range of motion were consistently observed. Radiographic evaluation confirmed bilateral hip OA, with Orthopedic Foundation for Animals (OFA) hip scores ranging from mild to severe [36]. The inclusion criteria required dogs to be older than 2 years, weigh more than 20 kg, and have not received any medications or nutritional supplements for at least six weeks before enrollment. Animals with concurrent orthopedic, neurologic, or systemic diseases were excluded from the study. All animals were fed the same commercially available dry food, housed in similar conditions, and maintained active work and activity levels throughout the study duration. Due to the presence of clinical OA signs in all enrolled dogs, a placebo group was not included for ethical reasons. However, the treatments under investigation have previously been evaluated in placebo-controlled studies [33,37].

### 2.1. Procedures

After selection, patients were randomly assigned to one of three groups: a PRP group (PRPG, n = 10), a PBMT group (PBMTG, n = 10), or a combined therapies group (PRP + PBMTG, n = 10). Since all animals had bilateral hip OA, both joints of each patient received the same treatment [33,37]. Dogs in the PRPG received two intra-articular (IA) injections of 2 mL of platelet-rich plasma (PRP) per hip joint. The PRP was prepared using the commercially available CRT PurePRP^®^ Kit (Companion Animal Health by Enovis^®^, Lewisville, TX, USA). The first injection was administered on Day 0, followed by a second injection 14 days later, as per the manufacturer’s guidelines. The preparation protocol was as previously described in our prior protocol [24]. For PRP preparation, 50 mL of whole blood was collected from the jugular vein into a 60 mL syringe preloaded with 10 mL of Anticoagulant Citrate Dextrose Solution. The blood was then transferred into a concentrating device and centrifuged at 3600 rpm for 1 min using the Executive Series Centrifuge II (Companion Animal Health by Enovis^®^, Lewisville, TX, USA). Following this initial centrifugation, the buffy coat and platelet-poor plasma were collected and transferred to a second concentrating device. A second centrifugation was performed at 3800 rpm for 5 min. After the second spin, the remaining platelet-poor plasma was reduced to a final volume of 4 mL. The device was gently agitated to resuspend the platelets, and the resulting PRP was aspirated into a 12 mL syringe. The PRP was administered immediately after preparation, without the use of an activating agent. PRP and whole blood samples were submitted to an external laboratory for compositional analysis and comparison.

IA administration was performed under light sedation, achieved through the simultaneous intravenous administration of medetomidine (0.01 mg/kg) and butorphanol (0.1 mg/kg). The procedure for hip joint injection has been previously described [9]. The animal was positioned in lateral recumbency, with the target joint facing upward. A 10 × 10 cm area centered over the greater trochanter was clipped and aseptically prepared. An assistant maintained the limb in a neutral position, parallel to the table. A 21-gauge, 2.5-inch needle was inserted just dorsal to the greater trochanter, perpendicular to the limb’s long axis, until the joint space was reached. Correct needle placement was confirmed by aspiration of synovial fluid.

The PBMTG received PBMT using a Class IV therapeutic laser (CTS-DUO, Companion Animal Health by Enovis^®^, Lewisville, TX, USA). Hair was clipped for blinding purposes, and no sedation was required during the procedure. Treatment sessions were administered over three consecutive weeks, with three sessions on alternate days in week 1, two sessions spaced two days apart in week 2, and one session in week 3. Following this initial treatment phase, a single maintenance session was performed monthly during follow-up evaluations. The PBMT parameters, detailed in Table 1, were selected based on the manufacturer’s recommendations and prior evidence supporting therapeutic efficacy [24,33].

Animals in the PRP + PBMTG received both therapies concurrently, following the same treatment schedules as the PRPG and PBMTG. A three-day rest period was prescribed for all groups after the days of the IA administrations. For blinding purposes, all treatments were conducted in a separate room, without the presence of the handlers. All animals had the same 10 × 10 cm area centered over the greater trochanter clipped. Animals in all groups were requested to come to the clinic on the same moments as PRP + PBMTG.

**Figure 1:**
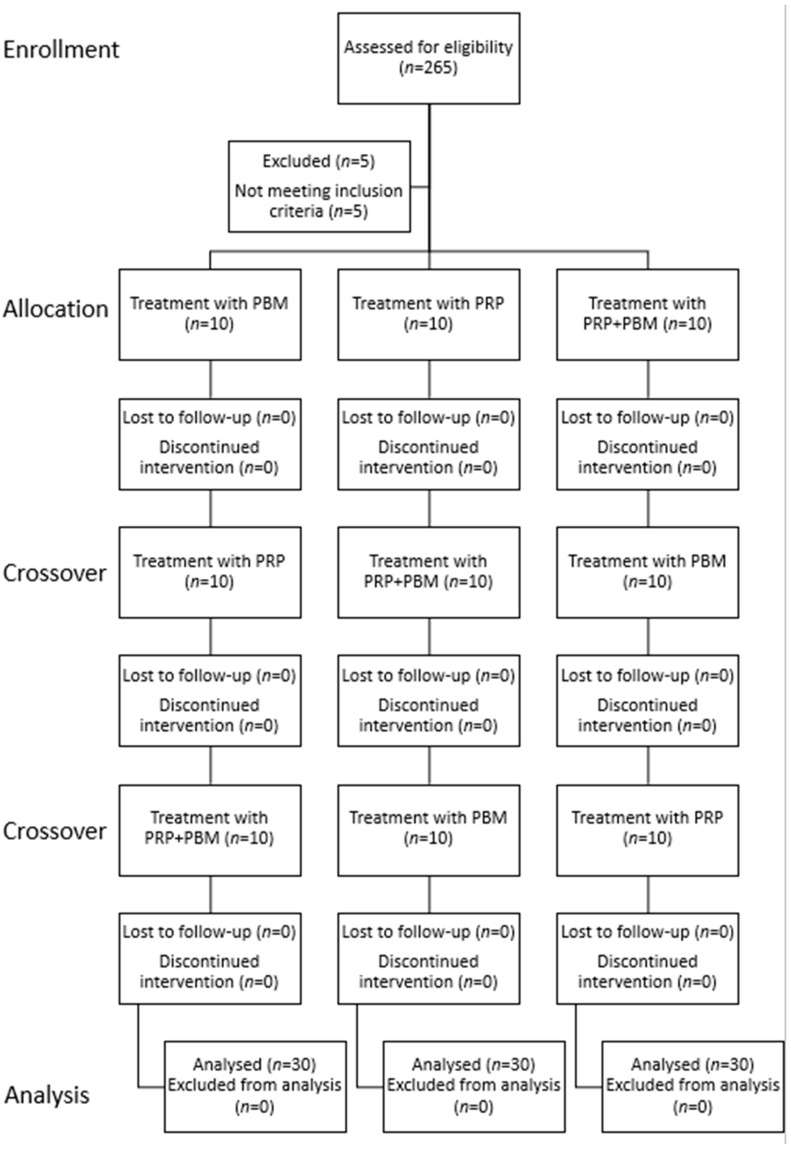
Full study flow diagram.

Follow-up evaluations were conducted at predetermined time points, on days 14 (+14 d, before the second IA PRP administration), 30 (+30 d), 60 (+30 d), 90 (+90 d), 120 (+120 d), 150 (+150 d), and 180 (+180 d) after the initial treatment. After a two-week rest period, the groups were crossed over, and the procedures were repeated. Upon completion of the second treatment cycle, a second crossover was performed, followed by another repetition of the procedures. This design ensured that all animals received each of the treatment protocols. A detailed study flow diagram is presented in Figure 1.

### 2.2. Outcome Measures

At each follow-up time point, a weight-bearing evaluation was performed using the Companion Stance Analyzer (Enovis^®^, Newark, DE, USA), following the procedure previously described [38]. The equipment was positioned in the center of a room, at least one meter from the walls. After calibration, dogs were encouraged to stand on the platform with one paw placed on each quadrant. A minimum of 20 measurements was recorded per animal, and the mean value was calculated. Since all animals in the study presented with bilateral disease, deviations from the normal weight-bearing distribution of the pelvic limbs were also assessed. This deviation was calculated by subtracting the measured combined pelvic limb weight-bearing (WB) from the expected normal value of 40% [39]. Additionally, a left-right symmetry index (SI) was calculated using the formula SI = [(WBR − WBL)/((WBR + WBL) × 0.5)] × 100, where WBR and WBL represent the weight-bearing values for the right and left pelvic limbs, respectively [40,41]. Negative values were made positive.

In addition to the weight-bearing distribution assessment, a digital version of the Canine Brief Pain Inventory (CBPI)—comprising the Pain Severity Score (PSS) and Pain Interference Score (PIS)—the Liverpool Osteoarthritis in Dogs (LOAD) questionnaire, and the Canine Orthopedic Index (COI)—which includes dimensions of stiffness, gait, function, and quality of life (QOL)—were completed sequentially by the same handler, who was blinded to the treatment group of each dog. All instruments used have been previously validated in Portuguese [42,43,44,45]. The occurrence of post-administration flares was recorded.

All procedures described were performed by the same researcher, who remained blinded to the treatment group of each dog throughout the study.

### 2.3. Statistical Analysis

Data were assessed for normality using the Shapiro–Wilk test. At each evaluation time point, group score variations were compared using the Mann–Whitney U test. The effect size was determined and considered to be small if r < 0.3, medium if 0.3 < r < 0.5, and large if r > 0.5. Demographic variables (age, sex, body weight, and breed) were recorded. Kaplan–Meier estimators were used to generate survival curves and estimate survival probabilities, with comparisons made using the log-rank test. For each outcome measure, specific events were defined based on previously established thresholds for clinically important changes in score. For the weight-bearing evaluation, a reduction of ≥2 in deviation and ≥10 in the SI was considered clinically relevant for dogs [46]. With the CBPI, a reduction of ≥1 in the Pain Severity Score (PSS) and ≥2 in the Pain Interference Score (PIS) was considered significant [47]. With the LOAD, a reduction of ≥4 was used as the event threshold [48,49]. With the COI, a reduction of ≥14 in the overall score; ≥4 in stiffness, gait, and function domains; and ≥3 in the QOL domain was considered [49]. The first and second occurrences of the events were evaluated. Patients whose scores exceeded baseline levels at the evaluation time point were censored. The Chi-squared test was performed to compare the frequency of post-administration joint flares between groups. All statistical analyses were performed using IBM SPSS Statistics, version 27. Statistical significance was set at *p* < 0.05.

## 3. Results

Animals of both sexes were included in the present sample (19 males and 11 females), with a mean age of 9.4 ± 2.7 years. All had an ideal body condition score for working dogs of 4/9 (n = 25) and 5/9 (n = 5) on the Laflamme scale [50], with a mean body weight of 26.6 ± 3.8 kg. Four dog breeds were present in the sample, similar to those found in most police and working dog populations worldwide: German Shepherd Dogs (n = 12), Belgian Malinois Shepherd Dogs (n = 7), Labrador Retrievers (n = 7), and Dutch Shepherd Dogs (n = 4). The dogs were used in drug detection and patrol work. Hips were graded with the OFA hip grading scheme, and 6 animals were classified as mild, 18 as moderate, and 6 as severe. All patients were followed up to the last evaluation moment after the final crossover. No additional treatment or medications were administered.

The composition of whole blood and PRP is presented in Table 2. Preparation of PRP took around 20 min, from the initial blood collection to the final administration.

**Figure 2:**
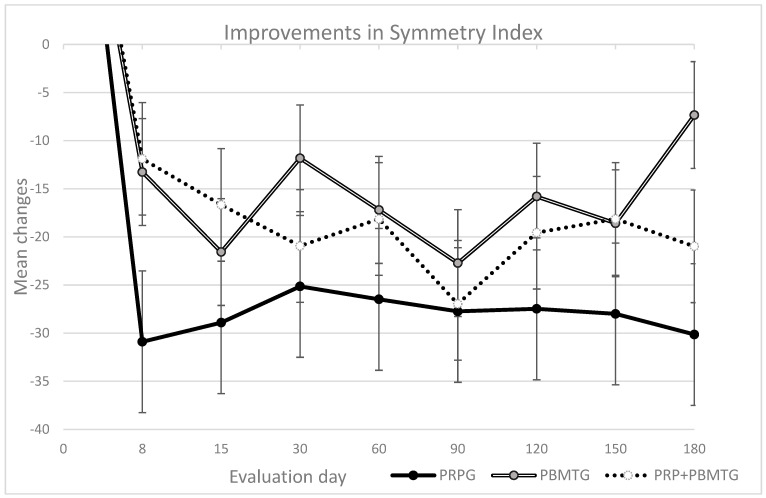
Mean change in Symmetry Index per group from baseline (±SEM) in the considered evaluation days. With the SI, a reduction of 10 or more is considered clinically significant.

**Figure 3:**
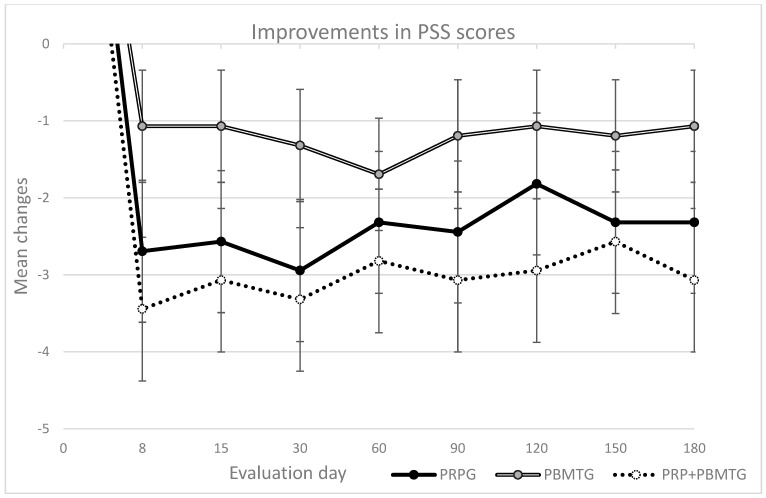
Mean change in Pain Severity Score (PSS) per group from baseline (±SEM) in the considered evaluation days. With the PSS, a reduction of 1 or more is considered clinically significant.

The results of the weight-bearing evaluation and scores of the different client-reported outcome measures in each group are presented in Table 3. No significant differences were observed on day 0. All treatments were able to produce clinically significant improvements in different evaluation modalities, although the degree of improvement varied in terms of magnitude and duration. The long-lasting improvements in objective measures (SI and deviation) as well as in pain levels (as measured with the CBPI) are particularly relevant, as pain and mobility impairment are among the major clinical signs in OA patients. The mean changes in SI and PSSs from baseline in each group are presented in Figure 2 and Figure 3, respectively. At the last follow-up, the combination of PRP and PBMT had a greater effect, showing a significant difference compared to the isolated treatments, with a moderate to large effect size. This finding was also observed with the LOAD score.

Table 4 and Table 5 present the results of the Kaplan–Meier estimators with each outcome measure, for a first and second occurrence of the set event (respectively). PRP + PBMTG showed more extended periods with better results, with scores taking longer to fall under a clinically significant level.

Post-administration joint flares between groups occurred in 50% of cases in PRPG and 30% of cases in PRP + PBMTG. * indicates significance when comparing groups. A significant difference was observed using the Chi-squared test (*p* < 0.01).

## 4. Discussion

OA significantly impacts the patient’s overall quality of life, as it produces pain and affects joint function and mobility [1,2,3,4]. Refractory chronic pain, impaired joint function, and mobility are associated with an increased risk of euthanasia in affected dogs [5,6]. To address the multiple dimensions of the disease and pain itself, clinicians typically prefer multimodal approaches in managing OA [51]. This approach enables the integration of the benefits of each therapeutic approach, thereby minimizing potential side effects. Our results show that the combined treatment with PRP and PBMT leads to greater and longer-lasting clinically significant improvements compared to the use of these treatments individually.

There are reports available on the use of PRP and PBMT in dogs with OA. However, the heterogeneity in products or equipment, administration and dosing protocols, and patient selection is reflected in the differences in results reported [52]. For PRP, clinical improvements have been recorded for 12 weeks [25,26,27] up to several months of improvements, as measured with clinical metrology instruments [9,21,22]. Multiple injection protocols have also been described, providing improvements in ROM, pain, lameness, and kinetics [28,29]. Clinical reports indicate that two PRP injections, administered 15 days apart, result in clinically significant improvements that last up to 6 months, compared to a placebo [30]. Similarly, PBMT has been shown to improve pain levels and lameness in dogs with OA, either as a single treatment or in combination with an NSAID [32,33]. The beneficial effects tend to wear off if treatment is discontinued [34] but show some persistence if a follow-up treatment protocol is established [23,30].

The results of this study showed that both PRP and PBMT were able to improve clinical scores and results in dogs with bilateral hip OA. The largest improvements were observed with CBPI and LOAD scores (lasting from 120 to 180 days). Since functional impairment and pain are some of the most significant clinical signs in OA patients, this finding is important. While some improvements were also observed with the COI and its dimensions, these were not as notable as those observed with the remaining evaluation modalities. One possibility justifying this finding is that the dimensions of the COI are designed to encompass a broader range of orthopedic conditions (the LOAD was specifically developed for OA patients, and the CBPI was initially validated in dogs with OA). Function and Gait, for example, while affected in OA patients, are often targeted with therapeutic exercise, something which was not introduced in this study. Another possibility is the fact that the animals included in this study are working dogs with an active lifestyle. Their natural work schedule may help target these dimensions without additional interventions if a pain management protocol is in place. Additionally, the scores of these dimensions were around mid-range, which leaves less room for improvement. In fact, including animals with higher initial client-reported outcome (CRO/CMI) scores increases the likelihood of detecting a clinically significant improvement, as there is more room for improvement to be observed and measured. In fact, some suggest that including animals with higher scores is preferable in clinical trials, making it easier to detect meaningful changes over time [52].

As hypothesized, the combined administration of PRP and PBMT resulted in consistently clinically significant improvements. The improvements were observed using both objective outcome measures and client-reported outcome measures, and persisted up to the last evaluation point, with a moderate to high effect size. These two modalities have historically been combined with other OA treatments. In human and dog OA reports, the concomitant use of PBMT with an NSAID has led to lower doses of NSAID being required to maintain an adequate response to treatment in dogs [33], or better results are achieved when PBMT is combined with therapeutic exercises [53], oral joint supplements [54], or biological treatments [55]. Similarly, PRP has been administered in combination with stem cells, showing added benefits [56]. We have observed similar results in a previous preliminary report [23,30]. It is not entirely clear whether these effects are the result of simply adding the benefits of two effective treatments or, more likely, the synergistic effect of the two. It has been shown that photoactivated PRP appears to shift the balance between pro- and anti-inflammatory cell signaling, in addition to achieving improved and prolonged growth factor release [57,58]. Additionally, by establishing a follow-up treatment protocol with PBMT, better control is obtained of the OA clinical signs, since this chronic disease of the joint will be present throughout the animal’s life.

Even though at least 80% of the cases of lameness and joint diseases in companion animals are classified as OA [59,60,61], there is growing evidence that suggests there are different OA phenotypes, reflecting different mechanisms of the disease [62]. These are also likely responsible for the varying responses to treatment observed in patients, reinforcing the need for a comprehensive evaluation of each patient. For example, it is well known that there is no direct correlation between clinical and radiographic signs [7] and that dogs with the same degree of OA can exhibit different compensation mechanisms [38]. That is a major interest for survival analysis, as it focuses on individual results, not just group results. We chose to evaluate the time for a first and second reduction below clinically significant improvement levels for several reasons. First, some patients may require more time to show significant improvements after treatment is started than the time to the first follow-up moment, which was conducted only 8 days after the start of treatment. A second reason is that if post-administration joint flares occur, these can affect the immediate follow-up moment. A final reason is that there is a natural variation in the clinical signs of OA. Since two of the treatment groups (PBMTG and PRP + PGMTG) received treatment sessions every month, it would be of interest to evaluate whether these sessions were sufficient to control these natural variations. The results of the Kaplan–Meier test show consistent improvements in all groups, similar to those observed in the between-group comparisons. The results also underscore the importance of regular monitorization of OA patients.

There are some side effects reported following PRP administration, which include injection pain and local inflammation. They are local, transient, and self-limiting, resolving within 2–10 days [63]. We did observe these side effects in some patients, which resolved without external intervention within 24–48 h. Our results showed that adding PBMT to the PRP administered led to fewer cases, which is an added benefit of the combined use.

This study has some limitations. The first is related to the nature of the animals included in the sample. Despite providing a homogeneous sample and around the age where OA is commonly diagnosed (with a mean age of 9.4 years), these were active police working dogs, meaning that clinical signs are usually detected early, and the animals are closely monitored throughout their lives. This is not always the case in the companion animal population. On the other hand, being active dogs, OA-related complaints may also be more noticeable. Future studies should include animals with a broader background. Additionally, we only included animals with bilateral hip OA. Response to treatment may differ if other joints are affected, either as a single joint or multiple joints. The lack of a placebo group is also a limitation, even though these treatments and parameters have previously been evaluated in placebo-controlled studies [33,37]. Finally, we need to consider the possible effect of continuous treatment. Although the full follow-up period covered two winters, the fact that the animals were being treated may have led to better outcomes after the first and second cross-overs. Still, if this is the case, it shows the importance of constant monitoring and management of the chronic disease that is OA, similar to other conditions, such as heart or kidney failure.

## 5. Conclusions

This study showed that PRP and PBMT could improve objective outcomes and client-reported outcome measures in dogs with OA. Their combined use leads to greater, longer-lasting, clinically significant improvements, likely due to a synergetic effect of the two treatment modalities. OA patients can benefit from close, individualized monitoring and ongoing disease management.

## Figures and Tables

**Table 1:** Photobiomodulation Therapy Treatment Parameters.

	Light Parameters (Dose)
**Wavelength (nm)**	980 nm
**Radiant Power (W)**	13
**Irradiance (W/cm^2^) at the skin surface**	2.6 (using a large contact treatment head)
**Fluence (J/cm^2^)**	15
**Total Joules**	5250
**Treatment Protocol**	Continuously moving grid pattern in contact over the treatment area at a speed of 2.5–7.5 cm/s, according to manufacturer recommendations.
**Treatment Area (cm^2^)**	350 (entire hip area)
**Treatment Time**	6 min, 44 s

**Table 2:** Mean values (±standard deviation) of whole blood and platelet-rich plasma product composition.

Parameter	Whole Blood		Platelet Concentrate	
	Mean Value	SD	Mean Value	SD
Platelets (×10^3^/mm^3^)	327.93	86.13	1708.53	440.99
RBC (×10^6^/mm^3^)	6.82	1.21	0.48	0.06
WDC (×10^3^/mm^3^)	11.18	4.36	4.47	3.40
Lymphocytes (×10^3^/mm^3^)	2.26	0.86	2.71	1.79
Monocytes (×10^3^/mm^3^)	0.76	0.41	0.57	0.35
Neutrophils (×10^3^/mm^3^)	7.73	3.49	0.85	0.35
Eosinophils (×10^3^/mm^3^)	0.42	0.47	0.35	0.41
Basophils (×10^3^/mm^3^)	0.01	0.02	0.00	0.00

**Table 3:** Variation in weight-bearing results and the considered client-reported outcome measures (median, inter-quartile range, and percentual change) by group and moment. CBPI—Canine Brief Pain Inventory; COI—Canine Orthopedic Index; CSI—Clinically significant improvement; LOAD—Liverpool Osteoarthritis in Dogs; PIS—Pain Interference Score; PSS—Pain Severity Score; QOL—Quality of Life. * indicates significance when comparing groups at each follow-up moment.

Measure		Group	T0				*p*		+8 d							*p*		ES		+15 d						*p*		ES		+30 d					*p*	ES		+60 d						*p*	ES
			Med	IQR					Med		IQR			Δ						Med		IQR	Δ							Med		IQR		Δ				Med		IQR		Δ			
**Weight bearing**	**Symmetry Index (CSI ≥ 10)**	PRPG	38.0	58.7			0.90		7.1		10.9			−30.9		0.03 *		0.16		9.1		10.9	−28.9			0.02 *		0.38		12.9		22.4		−25.1	0.03 *	0.30		11.5		28.7		−26.5		0.77	0.17
		PBMTG	31.8	39.6					18.6		23.5			−13.3						10.3		13.3	−21.6							20.0		46.3		−11.8				14.6		20.5		−17.2			
		PRP + PBMTG	32.1	39.6					20.2		27.8			−11.9						15.4		27.8	−16.7							11.1		17.5		−20.9				14.0		17.1		−18.1			
	**Deviation** **(CSI ≥ 2)**	PRPG	4.7	5.3			0.42		1.5		2.0			−3.2		0.08		0.28		2.0		2.0	−2.7			0.16		0.38		3.0		4.8		−1.7	0.85	0.20		2.5		4.8		−2.2		0.21	0.13
		PBMTG	7.3	10.0					2.0		3.8			−5.3						3.5		3.5	−3.8							3.5		5.8		−3.8				4.0		5.8		−3.3			
		PRP + PBMTG	6.0	6.7					2.5		2.8			−3.5						2.0		3.0	−4.0							3.0		4.0		−3.0				3.0		3.0		−3.0			
**CBPI**	**PSS (range 0–10; CSI ≥ 1)**	PRPG	5.8	2.2			0.11		3.1		3.8			−2.7		0.17		0.10		3.3		3.9	−2.6			0.30		0.38		2.9		3.5		−2.9	0.01 *	0.41		3.5		3.6		−2.3		0.51	0.07
		PBMTG	5.3	1.8					4.3		3.8			−1.1						4.3		2.0	−1.1							4.0		2.2		−1.3				3.6		2.2		−1.7			
		PRP + PBMTG	5.3	3.0					1.9		2.5			−3.4						2.3		3.8	−3.1							2.0		3.4		−3.3				2.5		3.9		−2.8			
	**PIS (range 0–10; CSI ≥ 2)**	PRPG	5.5	3.8			0.24		2.7		5.0			−2.8		0.18		0.15		3.4		5.0	−2.1			0.77		0.38		2.6		4.3		−2.9	0.16	0.21		3.6		3.8		−1.9		0.69	0.12
		PBMTG	5.8	4.1					4.0		2.9			−1.8						4.0		2.6	−1.8							3.9		3.9		−1.9				4.0		3.2		−1.8			
		PRP + PBMTG	4.4	4.7					1.9		3.7			−2.5						1.9		5.2	−2.5							2.0		4.0		−2.4				2.0		4.7		−2.4			
**LOAD (range 0–52; CSI ≥ 4)**		PRPG	22.6	14.0			0.46		14.0		9.5			−8.6		0.01 *		0.43		14.0		11.0	−8.6			0.03 *		0.38		14.0		10.8		−8.6	0.04 *	0.45		18.5		13.8		−4.1		0.04 *	0.38
		PBMTG	27.9	17.0					21.5		14.8			−6.4						22.5		14.5	−5.4							22.5		17.5		−5.4				21.0		18.5		−6.9			
		PRP + PBMTG	26.6	15.0					13.0		13.5			−13.6						13.5		16.3	−13.1							15.0		15.3		−11.6				16.0		13.8		−10.6			
**COI**	**Stiffness (range 0–16; CSI ≥ 4)**	PRPG	10.6	4.0			0.44		4.0		4.0			−6.6		0.19		0.01		4.5		4.0	−6.1			0.52		0.38		4.0		2.8		−6.6	0.04 *	0.35		4.5		4.0		−6.1		0.67	0.19
		PBMTG	10.6	4.0					6.0		4.0			−4.6						7.0		4.0	−3.6							7.0		4.0		−3.6				5.5		4.0		−5.1			
		PRP + PBMTG	9.3	5.3					4.0		5.8			−5.3						4.0		5.5	−5.3							4.0		4.0		−5.3				4.5		4.0		−4.8			
	**Function (range 0–16; CSI ≥ 4)**	PRPG	9.3	5.3			0.27		4.0		5.0			−5.3		0.02 *		0.47		4.0		3.8	−5.3			0.45		0.38		4.0		3.8		−5.3	0.02 *	0.43		4.0		5.0		−5.3		0.04 *	0.31
		PBMTG	9.3	4.7					6.5		5.8			−2.8						6.5		6.5	−2.8							6.5		5.8		−2.8				6.0		5.8		−3.3			
		PRP + PBMTG	8.6	4.0					4.0		6.8			−4.6						4.0		7.3	−4.6							4.0		5.5		−4.6				4.0		4.0		−4.6			
	**Gait (range 0–20; CSI ≥ 4)**	PRPG	10.6	4.0			0.29		6.0		5.8			−4.6		0.02 *		0.61		7.0		5.8	−3.6			0.33		0.38		5.0		5.8		−5.6	0.15	0.26		8.0		5.8		−2.6		0.56	0.08
		PBMTG	12.6	6.3					9.0		6.8			−3.6						9.0		6.8	−3.6							9.0		9.5		−3.6				9.0		6.0		−3.6			
		PRP + PBMTG	10.6	8.6					5.0		7.5			−5.6						6.0		9.8	−4.6							5.5		7.8		−5.1				8.0		8.8		−2.6			
	**QOL (range 0–12; CSI ≥ 3)**	PRPG	6.7	4.0			0.76		5.0		4.0			−1.7		0.22		0.16		6.0		3.8	−0.7			0.04 *		0.38		4.0		3.0		−2.7	0.04 *	0.50		5.5		4.0		−1.2		0.02 *	0.34
		PBMTG	8.0	4.0					4.0		3.8			−4.0						4.0		4.5	−4.0							4.0		4.8		−4.0				4.0		4.0		−4.0			
		PRP + PBMTG	6.0	6.3					3.0		5.0			−3.0						3.5		5.0	−2.5							4.5		4.8		−1.5				4.0		4.0		−2.0			
	**Overall (range 0–64; CSI ≥ 14)**	PRPG	37.2	17.3			0.39		19.0		18.8			−18.2		0.70		0.24		21.5		17.3	−15.7			0.53		0.38		17.0		15.3		−20.2	0.32	0.13		22.0		18.8		−15.2		0.71	0.11
		PBMTG	40.6	19.0					25.5		20.3			−15.1						26.5		21.8	−14.1							26.5		24.0		−14.1				24.5		19.8		−16.1			
		PRP + PBMTG	34.6	24.3					16.0		25.0			−18.6						17.5		27.5	−17.1							18.0		22.0		−16.6				20.5		20.8		−14.1			
**Measure**		**Group**	**+90 d**							***p***		**ES**			**+120 d**						***p***			**ES**			**+150 d**						***p***		**ES**		**+180 d**						***p***		**ES**
			**Med**			**IQR**		**Δ**							**Med**		**IQR**		**Δ**								**Med**		**IQR**		**Δ**						**Med**		**IQR**		**Δ**				
**Weight bearing**	**Symmetry Index (CSI ≥ 10)**	PRPG	10.3		28.6			−27.7		0.29			0.22		10.5		15.4		−27.5		0.02 *				0.05		10.0		21.8		−28.0		0.47		0.23		7.9		25.6		−30.1		0.01 *		0.82
		PBMTG	9.1		14.9			−22.7							16.0		26.9		−15.8								13.2		20.1		−18.6						24.5		33.5		−7.3				
		PRP + PBMTG	5.1		14.0			−27.0							12.5		16.9		−19.6								14.0		19.7		−18.1						11.1		13.1		−21.0				
	**Deviation** **(CSI ≥ 2)**	PRPG	3.5		6.0			−1.2		<0.01 *			0.56		2.0		3.0		−2.7		0.01 *				0.22		2.0		3.8		−2.7		0.07		0.16		2.5		4.0		−2.2		0.05 *		0.42
		PBMTG	4.5		4.0			−2.8							3.0		4.0		−4.3								3.5		4.8		−3.8						5.0		6.0		−2.3				
		PRP + PBMTG	1.0		4.0			−5.0							3.0		3.0		−3.0								3.0		3.0		−3.0						2.5		3.0		−3.5				
**CBPI**	**PSS (range 0–10; CSI ≥ 1)**	PRPG	3.4		4.0			−2.4		0.04 *			0.33		4.0		3.4		−1.8		0.03 *				0.39		3.5		3.4		−2.3		0.04 *		0.34		3.5		3.2		−2.3		0.04 *		0.40
		PBMTG	4.1		1.9			−1.2							4.3		2.4		−1.1								4.1		1.9		−1.2						4.3		2.6		−1.1				
		PRP + PBMTG	2.3		4.0			−3.1							2.4		3.9		−2.9								2.8		3.9		−2.6						2.3		3.9		−3.1				
	**PIS (range 0–10; CSI ≥ 2)**	PRPG	3.0		4.3			−2.5		0.53			0.29		4.1		3.7		−1.4		0.32				0.18		3.1		4.2		−2.4		0.56		0.07		3.6		4.1		−1.9		0.62		0.11
		PBMTG	3.9		2.1			−1.9							4.0		2.4		−1.8								4.0		2.7		−1.8						3.9		2.5		−1.9				
		PRP + PBMTG	2.3		4.2			−2.1							2.1		4.2		−2.3								2.6		4.1		−1.8						2.2		4.2		−2.2				
**LOAD (range 0–52; CSI ≥ 4)**		PRPG	18.5		10.8			−4.1		0.04 *			0.92		19.0		11.8		−3.6		0.04 *				0.95		18.5		12.5		−4.1		0.04 *		0.99		20.5		12.0		−2.1		0.04 *		0.55
		PBMTG	23.0		14.5			−4.9							23.0		19.3		−4.9								24.0		17.5		−3.9						24.0		16.8		−3.9				
		PRP + PBMTG	17.0		13.8			−9.6							16.5		15.5		−10.1								18.0		15.5		−8.6						18.0		15.5		−8.6				
**COI**	**Stiffness (range 0–16; CSI ≥ 4)**	PRPG	5.0		4.0			−5.6		0.53			0.08		5.5		4.0		−5.1		0.03 *				0.39		6.5		4.8		−4.1		0.02 *		0.31		4.5		3.8		−6.1		0.03 *		0.43
		PBMTG	7.0		4.0			−3.6							8.0		4.0		−2.6								8.0		6.8		−2.6						7.5		4.0		−3.1				
		PRP + PBMTG	4.0		5.8			−5.3							4.5		6.0		−4.8								4.5		6.0		−4.8						4.5		6.0		−4.8				
	**Function (range 0–16; CSI ≥ 4)**	PRPG	6.0		4.0			−3.3		0.03 *			0.10		6.5		4.0		−2.8		0.02 *				0.42		6.0		4.0		−3.3		0.02 *		0.34		6.0		4.0		−3.3		0.02 *		0.33
		PBMTG	7.0		4.5			−2.3							7.0		5.8		−2.3								7.0		5.8		−2.3						7.0		5.8		−2.3				
		PRP + PBMTG	4.0		5.8			−4.6							4.0		4.8		−4.6								4.0		4.8		−4.6						4.0		5.5		−4.6				
	**Gait (range 0–20; CSI ≥ 4)**	PRPG	7.5		5.8			−3.1		0.47			0.24		8.5		5.5		−2.1		0.21				0.19		8.0		5.3		−2.6		0.16		0.03		8.0		5.0		−2.6		0.21		0.07
		PBMTG	9.0		5.8			−3.6							10.0		7.8		−2.6								10.0		7.5		−2.6						9.5		7.5		−3.1				
		PRP + PBMTG	7.5		8.3			−3.1							8.0		7.0		−2.6								8.0		6.0		−2.6						8.0		7.5		−2.6				
	**QOL (range 0–12; CSI ≥ 3)**	PRPG	5.0		18.5			−1.7		0.04 *			0.05		6.0		4.0		−0.7		0.04 *				0.48		8.0		6.0		1.4		0.04 *		0.14		6.0		3.8		−0.7		0.04 *		0.32
		PBMTG	4.5		3.8			−3.5							5.0		3.8		−3.0								5.0		3.8		−3.0						5.0		3.8		−3.0				
		PRP + PBMTG	4.5		4.5			−1.5							4.0		3.8		−2.0								4.0		3.8		−2.0						4.5		3.8		−1.5				
	**Overall (range 0–64; CSI ≥ 14)**	PRPG	23.5		32.3			−13.7		0.59			0.11		26.5		17.5		−10.7		0.29				0.20		28.5		20.0		−8.7		0.02 *		0.35		24.5		16.5		−12.7		0.29		0.07
		PBMTG	27.5		18.0			−13.1							30.0		21.3		−10.6								30.0		23.8		−10.6						29.0		21.0		−11.6				
		PRP + PBMTG	20.0		24.3			−14.6							20.5		21.5		−14.1								20.5		20.5		−14.1						21.0		22.8		−13.6				

**Table 4:** Time (in days) to record a first reduction below clinically significant levels, calculated with Kaplan–Meier estimators and compared with the Log-rank test. * indicates significance.

Outcome Measure		Log Rank Test	PRPG				PBMTG				PRP + PBMTG			
			Mean	SD	95% CI		Mean	SD	95% CI		Mean	SD	95% CI	
Weight bearing	SI	0.03 *	94.7	14.8	65.6	123.7	68.3	14.2	40.4	96.1	121.6	15.2	89.2	133.0
	Deviation	0.02 *	95.6	16.8	62.7	128.6	79.9	15.7	49.3	110.6	162.1	14.9	123.0	154.4
CBPI	PSS	0.02 *	101.4	14.9	72.2	130.6	69.8	14.1	42.0	97.5	101.8	14.2	42.5	142.0
	PIS	0.04 *	126.1	13.6	99.5	152.7	113.9	15.5	83.5	144.3	133.7	14.8	63.1	175.9
LOAD		<0.01 *	126.3	12.2	102.3	150.3	112.1	13.9	84.8	139.4	156.7	14.5	79.6	197.9
COI	Stiffness	0.03 *	66.3	14.3	38.3	94.3	49.8	12.5	25.3	74.4	70.0	12.4	23.9	105.3
	Function	0.02 *	61.3	13.6	34.6	88.1	59.0	12.8	33.9	84.1	80.2	13.8	28.3	119.3
	Gait	0.01 *	93.1	13.6	66.6	119.7	72.9	13.9	45.6	100.4	105.9	14.4	44.7	146.9
	QOL	0.02 *	50.8	12.7	25.9	75.7	39.6	10.3	19.4	59.7	66.8	12.4	21.8	101.9
	Overall	0.04 *	73.5	14.3	45.5	101.5	66.2	13.5	39.7	92.7	96.7	7.9	51.0	119.3

**Table 5:** Mean change in Pain Severity Score (PSS) per group from baseline (±SEM) in the considered evaluation days. With the PSS, a reduction of 1 or more is considered clinically significant. * indicates significance when comparing groups.

Outcome Measure		Log Rank Test	PRPG				PBMTG				PRP + PBMTG			
			Mean	SD	95% CI		Mean	SD	95% CI		Mean	SD	95% CI	
Weight bearing	SI	0.03 *	118.5	13.9	91.1	145.9	96.5	13.3	70.4	122.6	129.0	7.5	100.0	129.3
	Deviation	0.02 *	101.0	12.5	76.4	125.6	103.0	12.5	78.6	127.4	178.3	11.8	102.9	169.9
CBPI	PSS	0.04 *	124	13.7	97.2	150.8	124.0	13.2	98.0	149.9	112.0	14.1	72.8	156.2
	PIS	0.04 *	119.0	13.5	92.6	145.7	103.0	14.5	74.6	131.4	147.0	14.4	58.7	180.0
LOAD		0.03 *	141.5	9.9	122.1	160.9	132.0	12.3	107.9	156.1	163.3	10.5	159.5	167.2
COI	Stiffness	0.03 *	68.5	13.0	42.9	94.0	64.0	12.8	38.9	89.0	81.0	14.2	53.3	108.7
	Function	0.03 *	73.0	12.8	47.9	98.1	79.5	11.8	56.4	102.6	112.0	9.9	92.4	131.6
	Gait	0.03 *	110.5	13.1	84.6	13.14	91.0	13.4	64.8	117.2	111.5	15.6	88.8	134.2
	QOL	0.02 *	87	14.3	59.0	114.9	92.5	14.6	83.4	98.1	107.5	11.8	84.4	130.6
	Overall	0.04 *	87.0	14.3	59.0	114.9	82.5	12.7	57.7	107.3	107.5	7.5	77.6	107.2

## Data Availability

All data generated or analyzed during this study are included in this published article. The data presented in this study are available upon reasonable request from the corresponding author, as they are the property of the Institution responsible for the animals and, by law, confidential.

## References

[1] Anderson K.L., O’Neill D.G., Brodbelt D.C., Church D.B., Meeson R.L., Sargan D., Summers J.F., Zulch H., Collins L.M. (2018). Prevalence, Duration and Risk Factors for Appendicular Osteoarthritis in a UK Dog Population under Primary Veterinary Care. Sci. Rep..

[2] Alves J.C., Santos A., Jorge P., Lavrador C., Carreira L.M. (2020). Clinical and Diagnostic Imaging Findings in Police Working Dogs Referred for Hip Osteoarthritis. BMC Vet. Res..

[3] Anderson K.L., Zulch H., O’Neill D.G., Meeson R.L., Collins L.M. (2020). Risk Factors for Canine Osteoarthritis and Its Predisposing Arthropathies: A Systematic Review. Front. Vet. Sci..

[4] Alves J.C.A., Jorge P.I.F., dos Santos A.M.M.P. (2022). A Survey on the Orthopedic and Functional Assessment in a Portuguese Population of Police Working Dogs. BMC Vet. Res..

[5] Pugliese M., Falcone A., Alibrandi A., Zirilli A., Passantino A. (2022). Risk Factors Regarding Dog Euthanasia and Causes of Death at a Veterinary Teaching Hospital in Italy: Preliminary Results. Vet. Sci..

[6] Pegram C., Gray C., Packer R.M.A., Richards Y., Church D.B., Brodbelt D.C., O’Neill D.G. (2021). Proportion and Risk Factors for Death by Euthanasia in Dogs in the UK. Sci. Rep..

[7] Enomoto M., de Castro N., Hash J., Thomson A., Nakanishi-Hester A., Perry E., Aker S., Haupt E., Opperman L., Roe S. (2024). Prevalence of Radiographic Appendicular Osteoarthritis and Associated Clinical Signs in Young Dogs. Sci. Rep..

[8] Alves J.C., Santos A., Jorge P., Lafuente P. (2022). A Multiple-Session Mesotherapy Protocol for the Management of Hip Osteoarthritis in Police Working Dogs. Am. J. Vet. Res..

[9] Alves J.C.C., Santos A., Jorge P., Lavrador C., Carreira L.M.M. (2021). Intraarticular Triamcinolone Hexacetonide, Stanozolol, Hylan G-F 20 and Platelet Concentrate in a Naturally Occurring Canine Osteoarthritis Model. Sci. Rep..

[10] Alves J.C., Santos A., Jorge P., Lafuente P. (2022). Multiple Session Mesotherapy for Management of Coxofemoral Osteoarthritis Pain in 10 Working Dogs: A Case Series. Can. Vet. J..

[11] Hunt J.R., Dean R.S., Davis G.N.D., Murrell J.C. (2015). An Analysis of the Relative Frequencies of Reported Adverse Events Associated with NSAID Administration in Dogs and Cats in the United Kingdom. Vet. J..

[12] Mabry K., Hill T., Tolbert M.K. (2021). Prevalence of Gastrointestinal Lesions in Dogs Chronically Treated with Nonsteroidal Anti-inflammatory Drugs. J. Vet. Intern. Med..

[13] Cachon T., Frykman O., Innes J.F., Lascelles B.D.X., Okumura M., Sousa P., Staffieri F., Steagall P.V., Van Ryssen B. (2023). COAST Development Group’s International Consensus Guidelines for the Treatment of Canine Osteoarthritis. Front. Vet. Sci..

[14] Mosley C., Edwards T., Romano L., Truchetti G., Dunbar L., Schiller T., Gibson T., Bruce C., Troncy E. (2022). Proposed Canadian Consensus Guidelines on Osteoarthritis Treatment Based on OA-COAST Stages 1–4. Front. Vet. Sci..

[15] Innes J.F., Clayton J., Lascelles B.D.X. (2010). Review of the Safety and Efficacy of Long-Term NSAID Use in the Treatment of Canine Osteoarthritis. Vet. Rec..

[16] Walton M.B., Cowderoy E.C., Wustefeld-Janssens B., Lascelles B.D.X., Innes J.F. (2014). Mavacoxib and Meloxicam for Canine Osteoarthritis: A Randomised Clinical Comparator Trial. Vet. Rec..

[17] Moreau M., Dupuis J., Bonneau N.H. (2003). Clinical Evaluation of a Nutraceutical, Carprofen and Meloxicam for the Treatment of Dogs with Osteoarthritis. Vet. Rec..

[18] Farrell M., Waibel F.W.A., Carrera I., Spattini G., Clark L., Adams R.J., Von Pfeil D.J.F., De Sousa R.J.R., Villagrà D.B., Amengual-Vila M. (2025). Musculoskeletal Adverse Events in Dogs Receiving Bedinvetmab (Librela). Front. Vet. Sci..

[19] Juhakoski R., Heliovaara M., Impivaara O., Kroger H., Knekt P., Lauren H., Arokoski J.P.A. (2008). Risk Factors for the Development of Hip Osteoarthritis: A Population-Based Prospective Study. Rheumatology.

[20] Frizziero A., Giannotti E., Ferraro C., Masiero S. (2012). Platelet Rich Plasma Intra-Articular Injections: A New Therapeutic Strategy for the Treatment of Knee Osteoarthritis in Sport Rehabilitation. A Systematic Review. Sport. Sci. Health.

[21] Alves J.C.A., dos Santos A.M.M.P., Jorge P.I.F., Lavrador C.F.T.V.B., Carreira L.M.A. (2021). Management of Osteoarthritis Using 1 Intra-Articular Platelet Concentrate Administration in a Canine Osteoarthritis Model. Am. J. Sports Med..

[22] Alves J.C., Santos A., Jorge P., Lavrador C., Carreira L.M. (2020). A Report on the Use of a Single Intra-Articular Administration of Autologous Platelet Therapy in a Naturally Occurring Canine Osteoarthritis Model—A Preliminary Study. BMC Musculoskelet. Disord..

[23] Alves J.C., Santos A., Carreira L.M. (2023). A Preliminary Report on the Combined Effect of Intra-Articular Platelet-Rich Plasma Injections and Photobiomodulation in Canine Osteoarthritis. Animals.

[24] Murray I.R., Geeslin A.G., Goudie E.B., Petrigliano F.A., LaPrade R.F. (2017). Minimum Information for Studies Evaluating Biologics in Orthopaedics (MIBO). J. Bone Jt. Surg..

[25] Arican M., Şimşek A., Parlak K., Atli K., Sönmez G. (2018). Matrix Metalloproteinases 2 and 9 Activity after Intra-Articular Injection of Autologous Platelet-Rich Plasma for the Treatment of Osteoarthritis in Dogs. Acta Vet. Brno.

[26] Fahie M.A., Ortolano G.A., Guercio V., Schaffer J.A., Johnston G., Au J., Hettlich B.A., Phillips T., Allen M.J., Bertone A.L. (2013). A Randomized Controlled Trial of the Efficacy of Autologous Platelet Therapy for the Treatment of Osteoarthritis in Dogs. J. Am. Vet. Med. Assoc..

[27] Silva R.F., Carmona J.U., Rezende C.M.F. (2013). Intra-Articular Injections of Autologous Platelet Concentrates in Dogs with Surgical Reparation of Cranial Cruciate Ligament Rupture. Vet. Comp. Orthop. Traumatol..

[28] Cook J.L., Smith P.A., Bozynski C.C., Kuroki K., Cook C.R., Stoker A.M., Pfeiffer F.M. (2016). Multiple Injections of Leukoreduced Platelet Rich Plasma Reduce Pain and Functional Impairment in a Canine Model of ACL and Meniscal Deficiency. J. Orthop. Res..

[29] Parlak K., Arican M. (2020). Effect of Intra-Articular Administration of Autologous PRP and Activated PRP on Inflammatory Mediators in Dogs with Osteoarthritis. Vet. Med..

[30] Alves J.C., Santos A., Jorge P. (2021). Platelet-Rich Plasma Therapy in Dogs with Bilateral Hip Osteoarthritis. BMC Vet. Res..

[31] Anders J., Kobiela Kertz A., Wu X., Riegel R.J., Goldbold J. (2017). Basic Principles of Photobiomodulation and Its Effects at the Cellular, Tissue, and System Levels. Laser Therapy in Veterinary Medicine: Photobiomodulation.

[32] Wardlaw J.L., Gazzola K.M., Wagoner A., Brinkman E., Burt J., Butler R., Gunter J.M., Senter L.H. (2019). Laser Therapy for Incision Healing in 9 Dogs. Front. Vet. Sci..

[33] Looney A.L., Huntingford J.L., Blaeser L.L., Mann S. (2018). A Randomized Blind Placebo-Controlled Trial Investigating the Effects of Photobiomodulation Therapy (PBMT) on Canine Elbow Osteoarthritis. Can. Vet. J..

[34] Alves J.C., Santos A., Jorge P., Carreira L.M. (2022). A Randomized Double-Blinded Controlled Trial on the Effects of Photobiomodulation Therapy in Dogs with Osteoarthritis. Am. J. Vet. Res..

[35] Alves J.C., Jorge P., Santos A. (2021). The Effect of Photobiomodulation Therapy on the Management of Chronic Idiopathic Large-Bowel Diarrhea in Dogs. Lasers Med. Sci..

[36] Alves J.C., Jorge P., Santos A. (2023). The Effect of Photobiomodulation Therapy on Inflammation Following Dental Prophylaxis. J. Vet. Dent..

[37] Flückiger M. (2008). Scoring Radiographs for Canine Hip Dysplasia—The Big Three Organisations in the World. Eur. J. Compagnion Anim. Pract..

[38] Alves J.C., Santos A., Jorge P., Lavrador C., Carreira L.M. (2022). Characterization of Weight-Bearing Compensation in Dogs With Bilateral Hip Osteoarthritis. Top. Companion Anim. Med..

[39] Clough W., Canapp S., Taboada L., Dycus D., Leasure C. (2018). Sensitivity and Specificity of a Weight Distribution Platform for the Detection of Objective Lameness and Orthopaedic Disease. Vet. Comp. Orthop. Traumatol..

[40] Walton M.B., Cowderoy E., Lascelles D., Innes J.F. (2013). Evaluation of Construct and Criterion Validity for the ‘Liverpool Osteoarthritis in Dogs’ (LOAD) Clinical Metrology Instrument and Comparison to Two Other Instruments. PLoS ONE.

[41] Volstad N., Sandberg G., Robb S., Budsberg S. (2017). The Evaluation of Limb Symmetry Indices Using Ground Reaction Forces Collected with One or Two Force Plates in Healthy Dogs. Vet. Comp. Orthop. Traumatol..

[42] Alves J.C., Santos A., Jorge P. (2022). Initial Psychometric Evaluation of the Portuguese Version of the Canine Brief Pain Inventory. Am. J. Vet. Res..

[43] Alves J.C., Jorge P., Santos A. (2022). Initial Psychometric Evaluation of the Portuguese Version of the Liverpool Osteoarthritis in Dogs. BMC Vet. Res..

[44] Alves J.C. (2023). Initial Psychometric Evaluation of the Portuguese Version of the Canine Orthopedic Index. Vet. Comp. Orthop. Traumatol..

[45] Alves J.C., Santos A., Jorge P., Lavrador C., Carreira L.M. (2022). Evaluation of Four Clinical Metrology Instruments for the Assessment of Osteoarthritis in Dogs. Animals.

[46] Alves J.C., Santos A., Lavrador C., Carreira L.M. (2023). Minimal Clinically Important Differences for a Weight Distribution Platform in Dogs with Osteoarthritis. Animals.

[47] Brown D.C., Bell M., Rhodes L. (2013). Power of Treatment Success Definitions When the Canine Brief Pain Inventory Is Used to Evaluate Carprofen Treatment for the Control of Pain and Inflammation in Dogs with Osteoarthritis. Am. J. Vet. Res..

[48] Innes J.F., Morton M., Lascelles B.D.X. Minimal Clinically-Important Difference for “Liverpool Osteoarthritis in Dogs” (LOAD) and Canine Orthopedic Index (COI). Proceedings of the 21st ESVOT Congress, ESVOT.

[49] Alves J.C., Innes J.F. (2023). Minimal Clinically-Important Differences for the “Liverpool Osteoarthritis in Dogs” (LOAD) and the “Canine Orthopedic Index” (COI) in Dogs with Osteoarthritis. PLoS ONE.

[50] Laflamme D. (1997). Development and Validation of a Body Condition Score System for Dogs. Canine Pr..

[51] Cai X., Zaki S. (2025). The Effect of Intra-articular Platelet-rich Plasma Injection on Pain and Lameness in Dogs with Osteoarthritis. Aust. Vet. J..

[52] Walton B., Cox T., Innes J. (2018). ‘How Do I Know My Animal Got Better?’—Measuring Outcomes in Small Animal Orthopaedics. In Pr..

[53] Vassão P.G., Parisi J., Penha T.F.C., Balão A.B., Renno A.C.M., Avila M.A. (2021). Association of Photobiomodulation Therapy (PBMT) and Exercises Programs in Pain and Functional Capacity of Patients with Knee Osteoarthritis (KOA): A Systematic Review of Randomized Trials. Lasers Med. Sci..

[54] Sanches M., Assis L., Criniti C., Fernandes D., Tim C., Renno A.C.M. (2018). Chondroitin Sulfate and Glucosamine Sulfate Associated to Photobiomodulation Prevents Degenerative Morphological Changes in an Experimental Model of Osteoarthritis in Rats. Lasers Med. Sci..

[55] Stancker T.G., Vieira S.S., Serra A.J., do Nascimento Lima R., dos Santos Feliciano R., Silva J.A., dos Santos S.A., dos Santos Vieira M.A., Simões M.C.B., Leal-Junior E.C. (2018). Can Photobiomodulation Associated with Implantation of Mesenchymal Adipose-Derived Stem Cells Attenuate the Expression of MMPs and Decrease Degradation of Type II Collagen in an Experimental Model of Osteoarthritis?. Lasers Med. Sci..

[56] Armitage A.J., Miller J.M., Sparks T.H., Georgiou A.E., Reid J. (2023). Efficacy of Autologous Mesenchymal Stromal Cell Treatment for Chronic Degenerative Musculoskeletal Conditions in Dogs: A Retrospective Study. Front. Vet. Sci..

[57] Irmak G., Demirtaş T.T., Gümüşderelioğlu M. (2020). Sustained Release of Growth Factors from Photoactivated Platelet Rich Plasma (PRP). Eur. J. Pharm. Biopharm..

[58] Carr B.J., Miller A.V., Colbath A.C., Peralta S., Frye C.W. (2024). Literature Review Details and Supports the Application of Platelet-Rich Plasma Products in Canine Medicine, Particularly as an Orthobiologic Agent for Osteoarthritis. J. Am. Vet. Med. Assoc..

[59] Lees P. (2003). Pharmacology of Drugs Used to Treat Osteoarthritis in Veterinary Practice. Inflammopharmacology.

[60] Venable R.O., Stoker A.M., Cook C.R., Cockrell M.K., Cook J.L. (2008). Examination of Synovial Fluid Hyaluronan Quantity and Quality in Stifle Joints of Dogs with Osteoarthritis. Am. J. Vet. Res..

[61] Johnston S.A. (1997). Osteoarthritis. Joint Anatomy, Physiology, and Pathobiology. Vet. Clin. N. Am. Small Anim. Pr..

[62] Vina E.R., Kwoh C.K. (2018). Epidemiology of Osteoarthritis. Curr. Opin. Rheumatol..

[63] Ornetti P., Nourissat G., Berenbaum F., Sellam J., Richette P., Chevalier X. (2016). Does Platelet-Rich Plasma Have a Role in the Treatment of Osteoarthritis?. Jt. Bone Spine.

